# Supplementary material for: Preparation and analysis of quinoa active protein (QAP) and its mechanism of inhibiting Candida albicans from a transcriptome perspective
Source: PeerJ. 2025 Feb 14;13:e18961. doi: 10.7717/peerj.18961 (PMC11831975; doi:10.7717/peerj.18961)
Supplement: Text S1 [file peerj-13-18961-s001.docx]

Operating Procedures:

1. **Dilution of Standards:** Dilute the standards provided in the kit.
2. **Sample Addition:** Set up blank wells (no samples and enzyme-labeled reagents are added to the blank control wells, and the rest of the operations are the same), standard wells, and sample wells to be tested. Add exactly 50 μL of the standard to the enzyme-labeled plate, and for the sample wells to be tested, first add 40 μL of the sample diluent, and then add 10 μL of the sample to be tested (the final dilution of the sample is 5 times).
3. **Incubation:** Seal the plate with a sealing film and incubate at 37 ℃ for 30 minutes.
4. **Solution Preparation:** Dilute the 30-fold concentrated washing solution 30 times with distilled water for later use.
5. **Washing:** Carefully remove the sealing film, discard the liquid, and dry by shaking. Fill each well with the washing solution, let stand for 30 seconds, discard the liquid, and repeat this process 5 times, and then pat dry.
6. **Enzyme Addition:** Add 50 μL of the enzyme-labeled reagent to each well, except for the blank wells.
7. **Incubation:** The operation is the same as in step 3.
8. **Washing:** The operation is the same as in step 5.
9. **Color Development:** First, add 50 μL of color developer A to each well, and then add 50 μl of color developer B. Gently shake to mix evenly, and develop color in the dark at 37°C for 10 minutes.
10. **Termination:** Add 50 μL of the stop solution to each well to terminate the reaction.
11. **Measurement:** Set the blank well to zero, and measure the absorbance (Optical Density (OD) value) of each well in sequence at a wavelength of 450 nm.
12. **Calculation:** The standard curve is generated by plotting the corresponding concentration on the horizontal (X) axis against the average Optical Density (OD) at 450 nm on the vertical (Y) axis. Calculate the mean OD value for each standard and sample.

 Standard Curve:

succinate dehydrogenase (SDH):

| Standard Concentration (U/L) | Mean OD450 |
| --- | --- |
| 320 | 1.7313 |
| 160 | 0.7810 |
| 80 | 0.4320 |
| 40 | 0.2239 |
| 20 | 0.1089 |
| 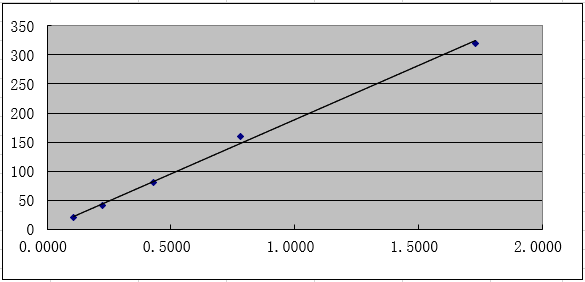 | |
| Y=1.8600 + 186.3595X  R^2^=0.9983 | |

Catalase (CAT):

| Standard Concentration (U/mL) | Mean OD450 |
| --- | --- |
| 16 | 1.8248 |
| 8 | 0.8130 |
| 4 | 0.4564 |
| 2 | 0.2472 |
| 1 | 0.1126 |
| 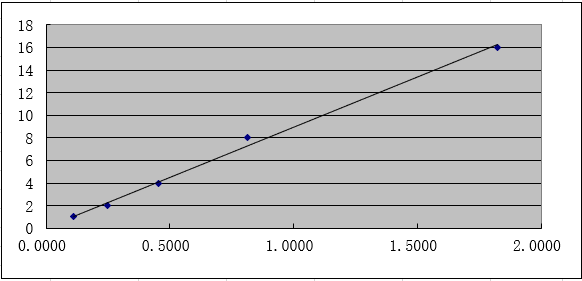 | |
| Y=0.0796 + 8.8601X  R^2^=0.9977 | |

Ca^2+^-Mg^2+^-ATPase:

| Standard Concentration (IU/L) | Mean OD450 |
| --- | --- |
| 80 | 1.7767 |
| 40 | 0.8136 |
| 20 | 0.4359 |
| 10 | 0.2300 |
| 5 | 0.1103 |
| 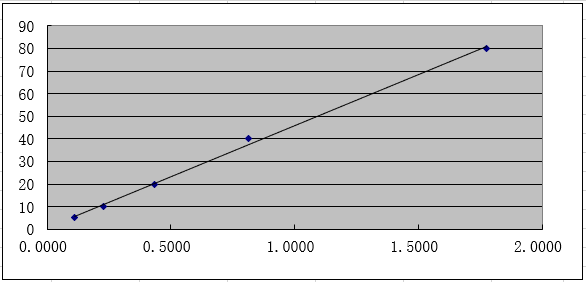 | |
| Y=0.4899 + 45.3169X  R^2^=0.9988 | |
