# Supplementary material for: Preparation and analysis of quinoa active protein (QAP) and its mechanism of inhibiting Candida albicans from a transcriptome perspective
Source: PeerJ. 2025 Feb 14;13:e18961. doi: 10.7717/peerj.18961 (PMC11831975; doi:10.7717/peerj.18961)
Supplement: Data S1 [file peerj-13-18961-s002.docx]

**Minimal Inhibitory Concentration of FLC:**


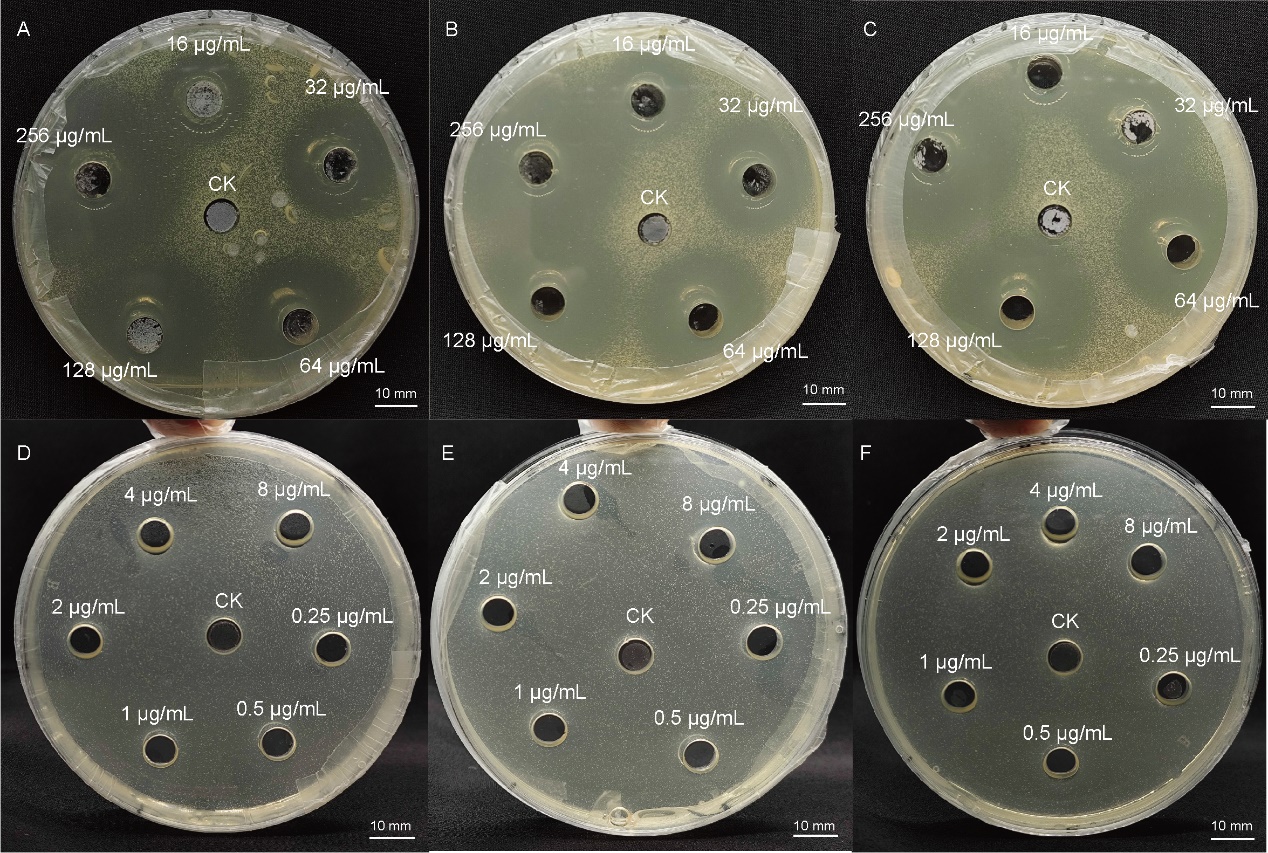


When the FLC concentration is 8 μg/mL, the antifungal effect is not evident. Thus, the MIC of FLC is considered to be 16 μg/mL.

**Minimal Inhibitory Concentration of QAP:**


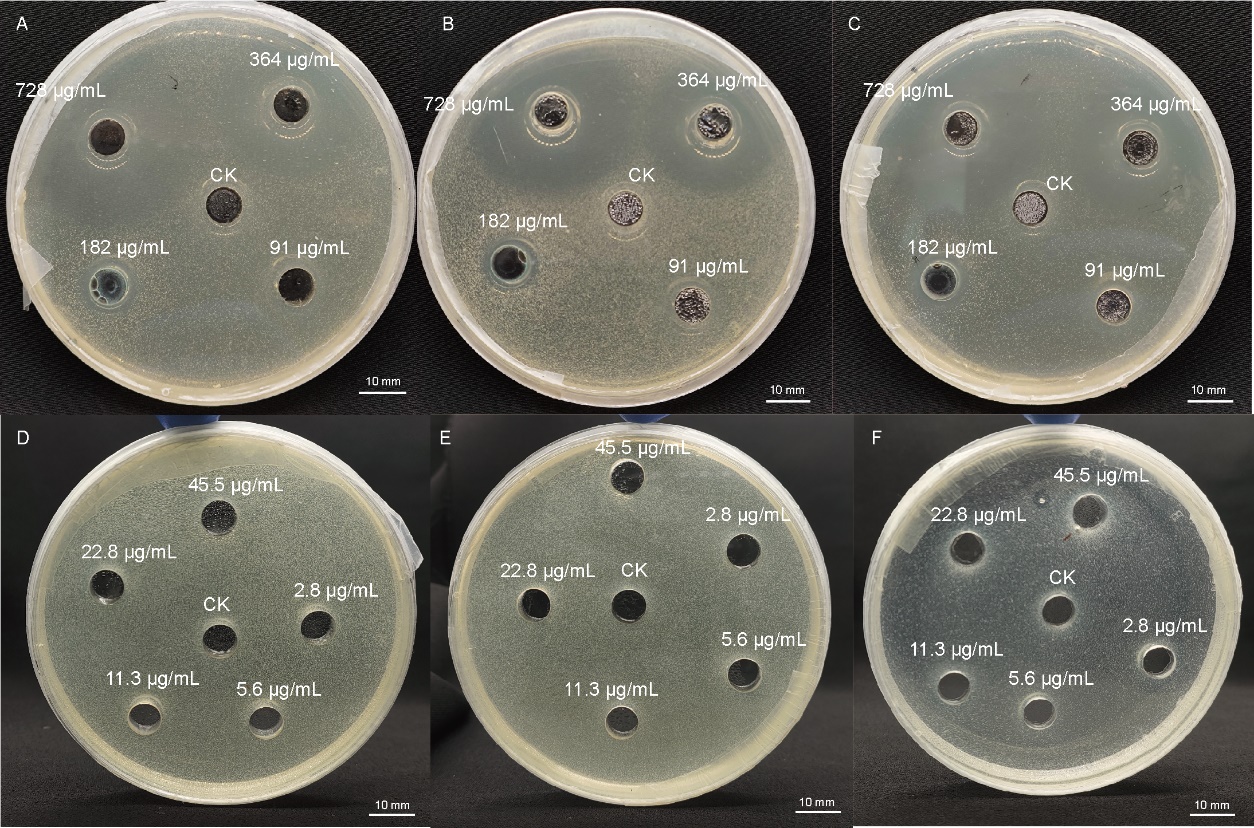


When the QAP concentration is 91 μg/mL, the antifungal effect is not evident. Thus, the MIC of QAP is considered to be 182 μg/mL.
