# Supplementary material for: Preparation and analysis of quinoa active protein (QAP) and its mechanism of inhibiting Candida albicans from a transcriptome perspective
Source: PeerJ. 2025 Feb 14;13:e18961. doi: 10.7717/peerj.18961 (PMC11831975; doi:10.7717/peerj.18961)
Supplement: Supplemental Information 19 [file peerj-13-18961-s019.docx]

**Sample**

Sample storage conditions and duration：2 days at -80℃

**Reverse transcription**

Complete reaction conditions

Step1. Genomic DNA removal

Prepare the following mixture in an RNase free centrifuge tube

| RNase-free ddH_2_O | to 10 μL |
| --- | --- |
| 5 × gDNA Buffer | 2 μL |
| RNA | ≤ 2μg |

Incubate at 42°C for three minutes, then place on ice until further use.

Step2. Prepare the first strand cDNA synthesis reaction solution

Prepare the following mixture in an RNase free centrifuge tube:

| Mix of Step1 |  |
| --- | --- |
| 10×King RT Buffer | 2 μL |
| FastKing RT Enzyme Mix | 1 μL |
| FQ-RT Primer Mix | 2 μL |
| RNase-free ddH_2_O | 5 μL |

Temperature and time

| 42°C | 15 min |
| --- | --- |
| 95°C | 3 min |

Storage conditions of cDNA: At -20℃

**qPCR TARGET INFORMATION**

Sequence accession number:

| ACT1 | CP017623 |
| --- | --- |
| PLB1 | CP017628 |
| SDH2 | CP017630 |
| ACO1 | CP017630 |
| MET6 | CP017630 |
| PCK1 | CP017630 |
| ALS4 | CP017628 |
| SIM1 | CP017623 |
| CST20 | CP017627 |
| FBA1 | CP017626 |
| PRA1 | CP017626 |
| ZRT1 | CP017626 |
| ERG1 | CP017623 |

Amplicons range from 80-150 bp

**qPCR protocol**

Perform RT-qPCR using the M5 Hiper SYBR Premix EsTaq with Tli RNaseH (Mei5 Biotechnology Co.,Ltd, China).

| cDNA (100 ng/μL) | 1 μL |
| --- | --- |
| Primer-F (10 μM) | 0.4μL |
| Primer-R (10 μM) | 0.4 μL |
| 2×M5 Hiper SYBR Premix EsTaq | 10 μL |
| RNase-free ddH_2_O | 8.2 μL |

**DATA ANALYSIS**

Each assay included three technical replicates to ensure the reliability of the results.
